# Supplementary figures and images for: MetaCRAST: reference-guided extraction of CRISPR spacers from unassembled metagenomes
Source: PeerJ. 2017 Sep 7;5:e3788. doi: 10.7717/peerj.3788 (PMC5592083; doi:10.7717/peerj.3788)

Number of false positive spacers

454

Illumina

AMD

EBPR

Tool

Crass

MetaCRAS

Minced

4

2

0

6

4

2

0

200

400

600

Read length

100

150

200

250

300

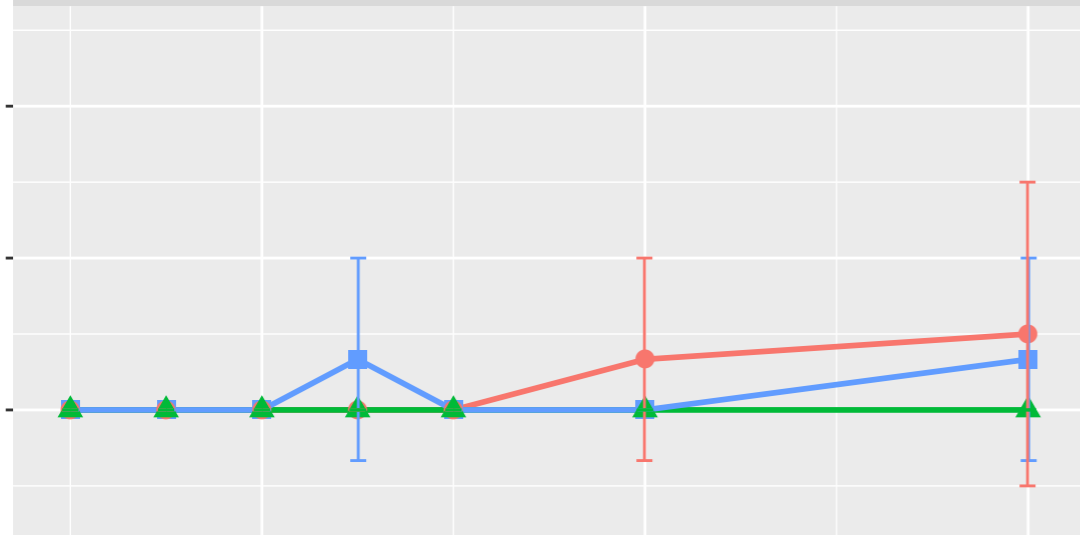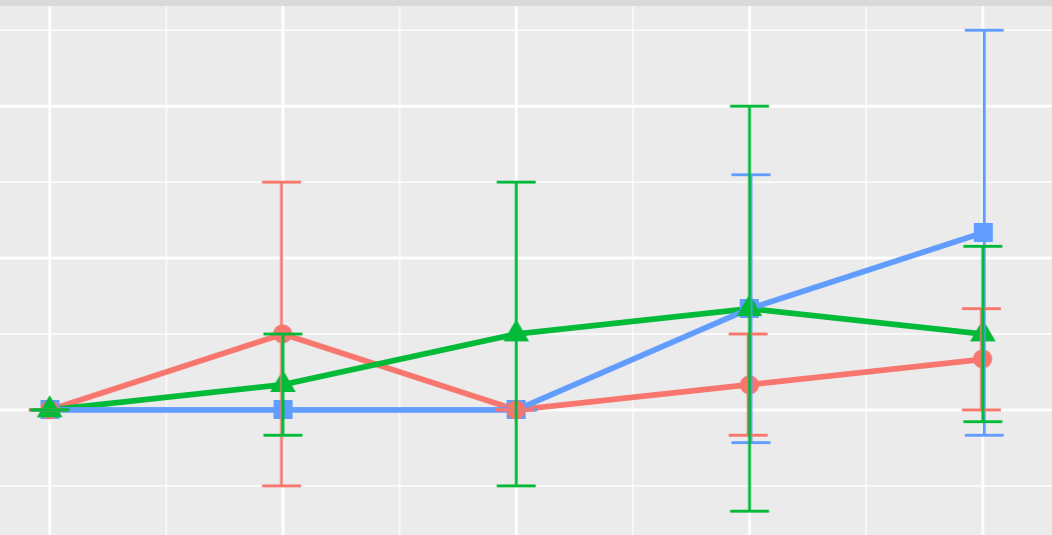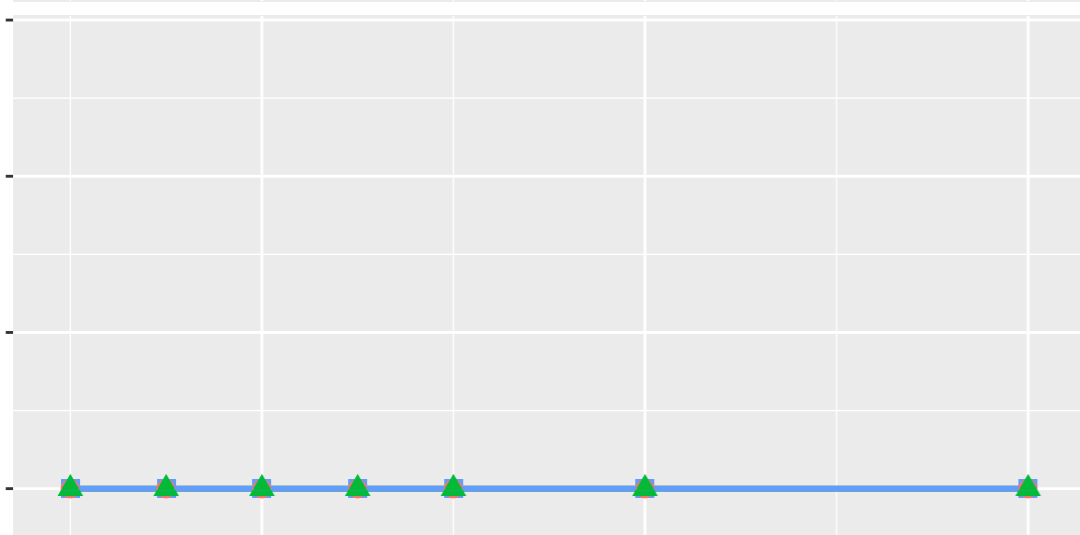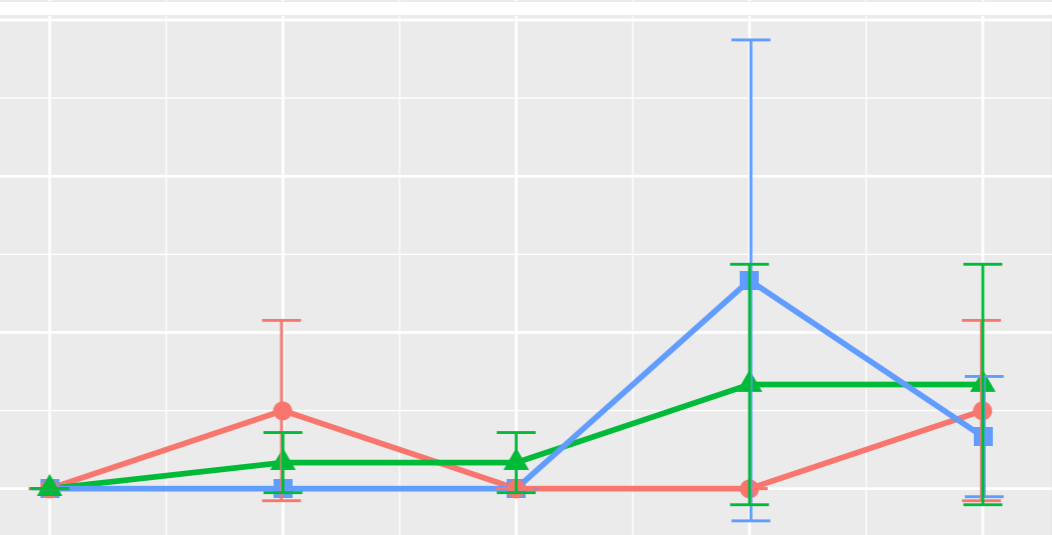

Supplement: Figure S1 — The procedure for generating the simulated metagenomes is described in Materials and Methods. The E-value threshold used for alignment of detected spacers against expected spacers was 1e−1. The number of detected spacers matching expected ones was subtracted from the total number of spacers detected to determine the number of false positive spacers for a particular method and condition. All data points represent the averages of three individual simulations and are presented with error bars representing two times the standard error above and two below the average. [file peerj-05-3788-s001.pdf]
