# Supplementary material for: MetaCRAST: reference-guided extraction of CRISPR spacers from unassembled metagenomes
Source: PeerJ. 2017 Sep 7;5:e3788. doi: 10.7717/peerj.3788 (PMC5592083; doi:10.7717/peerj.3788)
Supplement: Table S1 — All simulated metagenomes contained 100,000 reads. 454 metagenomes were generated with this command: grinder—reference_file AMDgenomes.fasta—abundance_file AMDprofile.txt -total_reads 100000 -read_dist (one of 100, 150, 200, 250, 300, 400, or 600) normal 50 -homopolymer_dist balzer. All 454 read length distributions were normal with a standard deviation of 50 bp. Illumina metagenomes were generated with this command: grinder -reference_file AMDgenomes.fasta -abundance_file AMDprofile.txt -total_reads 100000 -read_dist (one of 100, 150, 200, 250, or 300) -md poly4 3e−3 3.3e−8. All Illumina read length distributions were uniform with all reads having exactly the average read length. [file peerj-05-3788-s002.docx]

**Table S1:** Profile used to generate simulated AMD metagenomes using Grinder. All simulated metagenomes contained 100,000 reads. 454 metagenomes were generated with this command: grinder -reference_file AMDgenomes.fasta - abundance_file AMDprofile.txt -total_reads 100000 -read_dist [one of 100, 150, 200, 250, 300, 400, or 600] normal 50 -homopolymer_dist balzer. All 454 read length distributions were normal with a standard deviation of 50 bp. Illumina metagenomes were generated with this command: grinder -reference_file AMDgenomes.fasta -abundance_file AMDprofile.txt -total_reads 100000 -read_dist [one of 100, 150, 200, 250, or 300] -md poly4 3e-3 3.3e-8. All Illumina read length distributions were uniform with all reads having exactly the average read length.

| Taxon | GenBank accession (chromosome) | Relative abundance (%) |
| --- | --- | --- |
| *Leptospirillum* sp. Group II 'CF-1' | NZ_CP012147.1 | 65 |
| *Ferroplasma acidarmanus* fer1 | NC_021592.1 | 35 |
