# Supplementary material for: MetaCRAST: reference-guided extraction of CRISPR spacers from unassembled metagenomes
Source: PeerJ. 2017 Sep 7;5:e3788. doi: 10.7717/peerj.3788 (PMC5592083; doi:10.7717/peerj.3788)
Supplement: Table S4 — Direct repeats were obtained from CRISPRdb tables of CRISPRs detected in the listed microbial genomes. The Leptospirillum sp. Group IV ‘UBA BS’ direct repeat was detected in the listed assembly using CRISPRFinder and was not found in CRISPRdb. [file peerj-05-3788-s004.docx]

**Table S3:** Taxonomy-guided query used for real AMD metagenome. Direct repeats were obtained from CRISPRdb tables of CRISPRs detected in the listed microbial genomes. The *Leptospirillum* sp. Group IV 'UBA BS' direct repeat was detected in the listed assembly using CRISPRFinder and was not found in CRISPRdb.

| Taxon | Accession number | Query sequence |
| --- | --- | --- |
| *Ferroplasma acidarmanus* fer1 | NC_021592.1  (GenBank) | ATTTCAATTCCTATATGGAATTATTTTAAC |
| *Ferroplasma acidarmanus* fer1 | NC_021592.1  (GenBank) | GTGTTTAGTCTATCTATAAGGGTTTGAAAT |
| *Leptospirillum* sp. Group II 'CF-1' | NZ_CP012147.1  (GenBank) | GTATTCCCCACGTTCGTGGGGATGAACCG |
| *Leptospirillum* sp. Group IV 'UBA BS' | GCA_000496115.1 (Assembly) | GTTTTCCCCGCATGCGCGGGGGTGTTTCT |
