# Supplementary material for: MetaCRAST: reference-guided extraction of CRISPR spacers from unassembled metagenomes
Source: PeerJ. 2017 Sep 7;5:e3788. doi: 10.7717/peerj.3788 (PMC5592083; doi:10.7717/peerj.3788)
Supplement: Table S4 — Direct repeats were obtained from CRISPRdb tables of CRISPRs detected in the listed microbial genomes. [file peerj-05-3788-s005.docx]

**Table S4:** Taxonomy-guided query used for the real EBPR metagenome. Direct repeats were obtained from CRISPRdb tables of CRISPRs detected in the listed microbial genomes.

| Taxon | Accession number | Query sequence |
| --- | --- | --- |
| Candidatus *Accumulibacter phosphatis* clade IIA str. UW-1 | NC_013194.1  (GenBank) | GTCTCAATCCCTTTGATTTCAGGGCTGGTTACTGAC |
| Candidatus *Accumulibacter phosphatis* clade IIA str. UW-1 | NC_013194.1  (GenBank) | GTTTCCCCCGCGTCAGCGGGGATAGGCCC |
