# Supplementary material for: MetaCRAST: reference-guided extraction of CRISPR spacers from unassembled metagenomes
Source: PeerJ. 2017 Sep 7;5:e3788. doi: 10.7717/peerj.3788 (PMC5592083; doi:10.7717/peerj.3788)
Supplement: Table S5 — The AMD metagenome was downloaded from http://data.imicrobe.us/project/view/1, while the EBPR metagenome was downloaded from http://data.imicrobe.us/project/view/22. [file peerj-05-3788-s006.docx]

**Table S5:** Details about the AMD and EBPR metagenomes used for evaluation of the tools. The AMD metagenome was downloaded from <http://data.imicrobe.us/project/view/1>, while the EBPR metagenome was downloaded from <http://data.imicrobe.us/project/view/22>.

| Study site | Sequencing technology | Total reads | Average read length (bp) |
| --- | --- | --- | --- |
| Acid mine drainage (AMD) | Sanger | 319,166 | 1021 |
| Enhanced biological phosphorus removal (EBPR) | Sanger | 224,516 | 985 |
