# Supplementary material for: MetaCRAST: reference-guided extraction of CRISPR spacers from unassembled metagenomes
Source: PeerJ. 2017 Sep 7;5:e3788. doi: 10.7717/peerj.3788 (PMC5592083; doi:10.7717/peerj.3788)
Supplement: Table S6 — CAP3 assembled AMD reads into contigs with default parameters. CRISPR DRs were detected in CAP3 contigs using PILER-CR. The DRs clustered with a similarity threshold of 0.9 were then used to search the real AMD metagenome. [file peerj-05-3788-s007.docx]

**Table S6:** Assembly-guided query used for the real AMD metagenome. CAP3 assembled AMD reads into contigs with default parameters. CRISPR DRs were detected in CAP3 contigs using PILER-CR. The DRs clustered with a similarity threshold of 0.9 were then used to search the real AMD metagenome.

| PILER-CR DR information | DR sequence |
| --- | --- |
| Contig784[Array1;Pos=75] | CGGTTCATCCCCACGAACGTGGGGAATAC |
| Contig1409[Array2;Pos=26] | ATTTCAGAAAAACTAGTTAGTATGGAAG |
| Contig2393[Array5;Pos=904] | CTTTGAAACTTTCTAAATAAGATTCTAAC |
| Contig3459[Array7;Pos=1814] | GTTAGAATCTTATTTAGAAAGTTTCAAAGT |
| Contig3740[Array9;Pos=78] | CTTTCAATCCTATCAAGGTTCTATTTTTAC |
| Contig3754[Array10;Pos=255] | GTATTCCCCACGTTCGTGGGGATGAACCG |
| Contig3832[Array11;Pos=246] | GTTTAAAAAGCACTAGGTAGTATGGAAG |
| Contig3945[Array12;Pos=153] | GTTAAAATCGAACCTTAATAGGATTGAAAG |
| Contig3946[Array13;Pos=1080] | CTTTCAATCCTATTAAGGTTCGATTTTAAC |
| Contig4021[Array14;Pos=104] | GTAAAAATAGAACCTTGATAGGATTGAAAG |
| Contig4922[Array16;Pos=82] | ATTAGAAATATATCCTATAAGGAATTGATAC |
| Contig6764[Array22;Pos=263] | GTCTTCCCCACGCCCGTGGGGGTGTTTC |
| Contig7045[Array23;Pos=529] | GTAGTCCCCACGTATGTGGGGGTGAAGGG |
| Contig7402[Array24;Pos=53] | GTCTTAATCCCTTATTTATCAGGTCTTACCTTCGTTT |
| Contig8016[Array26;Pos=72] | GTGTTTAGTCTATCTATAAGGGTTTGAAAT |
| Contig8725[Array28;Pos=280] | GAAACACCCCCACGGGCGTGGGGAAGAC |
| Contig9087[Array29;Pos=204] | ATTTCCATAATAGAAATATTATGGCTCTATTGAAGC |
| Contig11151[Array31;Pos=153] | CTTCCATACTACCTAGTGCTTTTTAAAC |
