# Supplementary material for: MetaCRAST: reference-guided extraction of CRISPR spacers from unassembled metagenomes
Source: PeerJ. 2017 Sep 7;5:e3788. doi: 10.7717/peerj.3788 (PMC5592083; doi:10.7717/peerj.3788)
Supplement: Table S7 — CAP3 assembled EBPR reads into contigs with default parameters. CRISPR DRs were detected in CAP3 contigs using PILER-CR. The DRs clustered with a similarity threshold of 0.9 were then used to search the real EBPR metagenome. [file peerj-05-3788-s008.docx]

**Table S7:** Assembly-guided query used for the real EBPR metagenome. CAP3 assembled EBPR reads into contigs with default parameters. CRISPR DRs were detected in CAP3 contigs using PILER-CR. The DRs clustered with a similarity threshold of 0.9 were then used to search the real EBPR metagenome.

| PILER-CR DR information | DR sequence |
| --- | --- |
| Contig162[Array1;Pos=1038] | CTTTGAAGCTCGCCCCGATTTAGAGGGGATTAAGAC |
| Contig1390[Array2;Pos=112] | TTTCTAAGCCGCCATCACGGCGGCAAAC |
| Contig3577[Array4;Pos=288] | CGGTTCATCCCCACAGATACGGGGAACAC |
| Contig4649[Array8;Pos=1549] | GAGCGTGTCGTTGCCGGC |
| Contig4987[Array9;Pos=92] | CTGCCGTTATCCCTGATGCCGAAAGGCGTTGAGCAC |
| Contig5142[Array11;Pos=112] | GTTTGCCGCCGTGATGGCGGCTTAGAAA |
| Contig5455[Array12;Pos=1527] | CATTCTCCCAGCTAATTATGTTGGGAGTGGATTGAAACA |
| Contig6574[Array13;Pos=70] | GTTTCAATCCGCGCCCCTCGTTGCCGAGGGGCGATGC |
| Contig7071[Array14;Pos=54] | GTCTCAATCCCTTTGATTTCAGGGCTGGTTACTGAC |
| Contig7106[Array16;Pos=1039] | GGGCCTATCCCCGCTGACGCGGGGGAAAC |
| Contig8721[Array18;Pos=119] | GTCAGTAACCAGCCCTGAAATCAAAGGGATTGAGAC |
| Contig10087[Array20;Pos=540] | GTTCTCGCTCCCCGACTTCCTGAAGGGGATTAAGAC |
| Contig11041[Array22;Pos=71] | GTTGTGAATTGCTTTCAAATTCTTAAGTAACTTAGTTCTTGCACAAC |
| Contig11786[Array23;Pos=429] | GTTTCCCCCGCGTCAGCGGGGATAGGCC |
| Contig12588[Array27;Pos=221] | GTCACAAAGCCCTATTTACGGGCAGGGGTGACGGAC |
| Contig12822[Array28;Pos=605] | GTCGCCCGTCACTCCGGTGACGGGCGTGGATTGAAAC |
