# Supplementary material for: MetaCRAST: reference-guided extraction of CRISPR spacers from unassembled metagenomes
Source: PeerJ. 2017 Sep 7;5:e3788. doi: 10.7717/peerj.3788 (PMC5592083; doi:10.7717/peerj.3788)
Supplement: Table S8 [file peerj-05-3788-s009.docx]

**Table S8:** Taxonomic profile of the real AMD metagenome determined using MetaPhyler.

| Taxon | % Abundance | Depth of coverage | Number of reads | Similarity with reference |
| --- | --- | --- | --- | --- |
| Ferroplasma | 67.68 | 16.69 | 960 | 88.54 |
| Leptospirillum | 23.48 | 5.79 | 318 | 95.43 |
| Euryarchaeota{phylum} | 2.35 | 0.58 | 105 | 83.49 |
| Thermoplasmatales{order} | 2.03 | 0.5 | 59 | 85.97 |
| Actinobacteria{phylum} | 1.06 | 0.26 | 34 | 85.92 |
| Chlorobiaceae{family} | 0.76 | 0.18 | 28 | 88.91 |
| Fusobacteria{phylum} | 0.6 | 0.14 | 30 | 88.18 |
| Actinobacteria (class){class} | 0.38 | 0.09 | 19 | 86.93 |
| Proteobacteria{phylum} | 0.31 | 0.07 | 23 | 85.18 |
| Aquificae{phylum} | 0.24 | 0.06 | 10 | 88.09 |
| Deinococcus-Thermus{phylum} | 0.21 | 0.05 | 10 | 83.41 |
| Chlorobi{phylum} | 0.14 | 0.03 | 15 | 86.03 |
| Firmicutes{phylum} | 0.13 | 0.03 | 4 | 84.3 |
| Actinomycetales{order} | 0.13 | 0.03 | 8 | 88.6 |
| Bacteroidetes{phylum} | 0.09 | 0.02 | 8 | 84.46 |
| Delftia | 0.09 | 0.02 | 4 | 99.56 |
| Caulobacteraceae{family} | 0.08 | 0.02 | 2 | 94.3 |
| Chromatiales{order} | 0.03 | 0 | 4 | 85.45 |
| Deltaproteobacteria{class} | 0.02 | 0 | 1 | 88.89 |
| Nocardioidaceae{family} | 0.02 | 0 | 1 | 96.59 |
| Micrococcaceae{family} | 0.01 | 0 | 1 | 87.25 |
| Frankia | 0.01 | 0 | 1 | 91.46 |
| Crenarchaeota{phylum} | 0.01 | 0 | 2 | 84 |
| Thermoplasma | 0 | 0 | 1 | 89.74 |
| Thermotogae{phylum} | 0 | 0 | 1 | 87.32 |
| Veillonellaceae{family} | 0 | 0 | 1 | 91.55 |
