# Supplementary material for: MetaCRAST: reference-guided extraction of CRISPR spacers from unassembled metagenomes
Source: PeerJ. 2017 Sep 7;5:e3788. doi: 10.7717/peerj.3788 (PMC5592083; doi:10.7717/peerj.3788)
Supplement: Table S9 [file peerj-05-3788-s010.docx]

**Supplementary Table S9:** Taxonomic profile of the real EBPR metagenome determined using MetaPhyler.

| Taxon | % Abundance | Depth of coverage | Number of reads | Similarity with reference |
| --- | --- | --- | --- | --- |
| Candidatus Accumulibacter | 61.54 | 10.42 | 463 | 97.82 |
| Proteobacteria{phylum} | 8.78 | 1.48 | 149 | 83.93 |
| Xanthomonadaceae  {family} | 6.22 | 1.05 | 60 | 90.8 |
| Bacteroidetes{phylum} | 4.61 | 0.78 | 56 | 84.12 |
| Comamonadaceae  {family} | 1.49 | 0.25 | 11 | 92.36 |
| Gammaproteobacteria  {class} | 1.35 | 0.22 | 29 | 88.69 |
| Betaproteobacteria{class} | 1.31 | 0.22 | 19 | 87.49 |
| Flavobacteriaceae{family} | 1.21 | 0.2 | 8 | 90.22 |
| Alphaproteobacteria  {class} | 1.14 | 0.19 | 14 | 89.3 |
| Actinomycetales{order} | 0.99 | 0.16 | 8 | 88.53 |
| Burkholderiales{order} | 0.94 | 0.15 | 14 | 88.32 |
| Rhodobacteraceae  {family} | 0.92 | 0.15 | 6 | 90.84 |
| Verrucomicrobia{phylum} | 0.71 | 0.12 | 7 | 84.87 |
| Fibrobacteres{phylum} | 0.67 | 0.11 | 40 | 86.58 |
| Actinobacteria{phylum} | 0.6 | 0.1 | 13 | 85.71 |
| Pseudoxanthomonas | 0.59 | 0.1 | 3 | 98.9 |
| Flavobacterium | 0.57 | 0.09 | 4 | 93.04 |
| Rhizobiales{order} | 0.56 | 0.09 | 4 | 87.59 |
| Rhodobacterales{order} | 0.51 | 0.08 | 3 | 88.28 |
| Flavobacteriales{order} | 0.49 | 0.08 | 5 | 87.78 |
| Chloroflexi{phylum} | 0.48 | 0.08 | 3 | 86.54 |
| Acidovorax | 0.45 | 0.07 | 3 | 93.63 |
| Sphingomonadaceae  {family} | 0.41 | 0.06 | 3 | 92.46 |
| Shigella | 0.4 | 0.06 | 2 | 99.06 |
| Rhodocyclaceae{family} | 0.39 | 0.06 | 17 | 85.19 |
| Phyllobacteriaceae  {family} | 0.35 | 0.05 | 3 | 90.43 |
| Flavobacteria{class} | 0.29 | 0.04 | 2 | 88.47 |
| Firmicutes{phylum} | 0.21 | 0.03 | 7 | 86.69 |
| Ectothiorhodospiraceae  {family} | 0.18 | 0.03 | 2 | 85.99 |
| Beijerinckiaceae{family} | 0.18 | 0.03 | 2 | 90.27 |
| Alteromonadales{order} | 0.17 | 0.02 | 5 | 89.19 |
| Mesorhizobium | 0.15 | 0.02 | 2 | 89.46 |
| Magnetospirillum | 0.11 | 0.01 | 1 | 93.24 |
| Aeromonas | 0.09 | 0.01 | 2 | 99.37 |
| Cytophagaceae{family} | 0.09 | 0.01 | 2 | 90.65 |
| Acetobacteraceae{family} | 0.09 | 0.01 | 1 | 91.27 |
| Neisseriaceae{family} | 0.08 | 0.01 | 2 | 89.1 |
| Chlorobi{phylum} | 0.05 | 0 | 2 | 86.33 |
| Xanthomonas | 0.04 | 0 | 2 | 87.1 |
| Synergistetes{phylum} | 0.03 | 0 | 1 | 84.88 |
| Methylophilales{order} | 0.03 | 0 | 1 | 87.6 |
| Clostridiaceae{family} | 0.03 | 0 | 1 | 89.71 |
| Moraxellaceae{family} | 0.03 | 0 | 1 | 86.67 |
| Acinetobacter | 0.03 | 0 | 3 | 93.75 |
| Bacteroidales{order} | 0.03 | 0 | 2 | 88.43 |
| Caulobacter | 0.03 | 0 | 1 | 92.96 |
| Clostridia{class} | 0.03 | 0 | 1 | 90.48 |
| Cyanobacteria{phylum} | 0.02 | 0 | 2 | 85.71 |
| Photobacterium | 0.02 | 0 | 1 | 89.16 |
| Burkholderiaceae{family} | 0.02 | 0 | 1 | 89.23 |
| Campylobacter | 0.02 | 0 | 3 | 90.48 |
| Bacillales{order} | 0.02 | 0 | 2 | 87.4 |
| Roseovarius | 0.01 | 0 | 1 | 91.41 |
| Aurantimonadaceae  {family} | 0.01 | 0 | 1 | 93.55 |
